# Musical Heart Murmurs

**Published:** 1880-02

**Authors:** J. M. Da Costa

**Affiliations:** Professor of Practice of Medicine in Jefferson Medical College, Philadelphia


[From the Clinical News—Special Report.]

### MUSICAL HEART MURMURS.—A CLINICAL LECTURE.

BY J. M. DA COSTA, M. D.
(Professor of Practice of Medicine in Jefferson Medical College, Philadelphia.)

This man is a laborer, fifty-eight years of age, P. W—— by name. Twenty-six years ago he had an attack of acute articular rheumatism, which lasted three months. He has had two severe attacks since then and several light ones. In February, 1879, he began to be short of breath upon exertion. His face grew puffy and his legs swollen. There was no cough nor precordial pain; occasionally there was palpitation. He was at that time confined to bed for four weeks, the least exertion bringing on dyspnœa and faintness.

Eight weeks ago he was admitted to the hospital for disease of the heart. He left the wards soon afterward much improved, but came back within a few weeks, down again with dropsy and shortness of breath—utterly unable to do any work.

When I examined him I ascertained the following condition of things: Pulse very irregular, so much so that I found it difficult to determine how frequent it was—it ranged from 60 to 68. Now and then there was a halt, or else a series of regular beats, followed by a series of irregular beats. Physical examination showed that the impulse of the heart was moderately forcible and could be felt in two intercostal spaces. The area of percussion dullness I found to be increased, and then I came upon these curious physical signs. At or near the apex I could distinguish a murmur, chiefly systolic, but at the end of this murmur, and sometimes taking the place of the whole murmur, I heard a low, musical sound, which was transmitted to the aortic orifice, but which grew fainter and fainter as the apex was left. And then I noted another murmur following the systolic murmur, or, at least, preceding the next systole. There is, in fact, a double murmur, whose seat of greatest intensity is at or near the apex. Neither of these murmurs is transmitted into the arteries of the neck; neither of them is to be heard in the brachial artery. I can distinguish, as I listen now, a second indistinct normal sound at the base of the heart; it is more indistinct at the right than at the left base.

Sometimes the musical murmur is loud; sometimes it is a mere echo of the murmur at the end of the systole; sometimes there is nothing but a musical murmur to be heard. In any case it is strictly systolic.

We have very plainly an intricate cardiac case with a musical murmur.

As my assistant happens to have a sphygmograph in the room, we will see if this irregularity of the heart's action can be detected in the tracings of the radial pulse. Yes, here is the systole, and here the long, irregular diastole. Very few of the beats, as we have them mapped out on this slip of paper, are equal in length.

Musical murmurs in heart disease are rare. When they are as distinct as this murmur is, they are particularly rare. A musical murmur in heart disease is the easiest of all murmurs to recognize. It is so much like a cooing chest-râle that we are very apt to overlook the murmur and mistake what we hear for a breath-sound. This error may always be obviated by telling the patient to stop breathing, when all respiratory sounds will cease, while cardiac murmurs will persist; as I have done this upon several occasions, there is no doubt that the sound comes from the heart in this case.

These musical murmurs in the heart sometimes take the place of the whole murmur. At other times the murmur begins rough, but winds up musically. In other words, there are times in which the musical murmur takes the place of all other sounds, and other times when it is only a musical ending of the murmur which we hear.

These musical cardiac murmurs have given rise to a great deal of discussion. By some authorities they have been regarded as significant of some special form of disease of the heart. They have been thought to be invariably indicative of a contracted aortic orifice. Again, it has been held that this musical murmur can only be produced by rigidity of the valves, due to atheroma or some other cause, or by something vibrating in the current of the blood, such as a detached portion of the valve, or a fragment of heart muscle torn loose, or a vegetation springing from the valve.

With regard to the first point, I can tell you positively, and this case alone will serve as a standing proof of what I say, that *musical murmurs are not limited to cases of aortic stenosis.* On the other hand, the explanation which says that the murmur is due to something vibrating in the blood current is correct certainly in a vast majority of instances. In most cases when I distinguish this musical murmur, and particularly when I find it present in mitral disease and limited in the apex, I make the diagnosis that there is some portion of the valve, or something springing from the valve, vibrating in the blood current.

I shall not dwell at all upon the secondary diastolic murmur in this case, showing change at the orifice itself. But before dismissing the subject, since these cases are rare, I may refer to the statement made by some that a musical murmur may be the result of anemia. I cannot recall a single persistent cardiac musical murmur which was anemic. The statement is based upon a mistake. There are anemic musical murmurs heard sometimes in the vessels of the neck, but I am speaking of those cases where they take the place in part, or entirely, of all the abnormal heart sounds.

A musical murmur may be due either to narrowing of the orifice or to something flapping in the blood current. The explanation which limits it to cases of narrowing of the aortic orifice is, as already said, not correct. How are we to distinguish the musical murmur attending disease of the aortic orifice from what we have in this case? In disease of the aortic orifice the musical murmur is transmitted into the arterial tree; can be heard over the brachial, femoral and carotid arteries. You may remember, then, that this extension of the sound is always in favor of its aortic origin. There is no extension of the sound in this case.

Do these musical murmurs disappear in time? Are they always like chest-râles, or do they finally give way and grow rough and harsh?

I have had occasions to watch murmurs of this kind for years, and I have known them to disappear. One case in particular I give you, that of a patient of mine, a young lady from the western portion of the State, who pays a visit to this city every year, and whom I have had under my eye ever since she was eight years of age. She is now eighteen years old. Ten years ago she had most marked signs of organic heart disease, as evinced by a musical murmur. This murmur has been growing less and less each year, until the last time she was in the city I examined her closely and found that it had entirely gone. We are, therefore, supported in the assertion that a musical murmur may disappear in the process of time.

This brings me to a discussion of the question of treatment, and this is not so important a matter as it is to discover what particular state of the walls of the heart is associated with the valvular disease. Here we have associated with the mitral disease a moderate amount of dilated hypertrophy, or rather of dilatation, with but a slight increase in the muscular structure of the heart. The patient's heart is feeble and often struggling, while his pulse, as I have already pointed out, is at times singularly irregular.

The chief indication here is to strengthen and secure rest for the muscles of the heart. Our treatment has, therefore, consisted in the conjoined use of digitalis and belladonna, with the purpose of strengthening the ventricles. We have given gtt. ij of the fluid extract of digitalis, and gtt. j of the fluid extract of belladonna, thrice daily. Under this treatment the heart has grown stronger and its impulse become more regular. The patient's pulse is better and his color improving. The signs of disturbed pulmonary circulation have disappeared. The musical murmur, though still perceptible, is not so loud as it was at first.

